# Research on the Dynamical Behavior of Public Opinion Triggered by Rumor Based on a Nonlinear Oscillator Model

**DOI:** 10.3390/e25121614

**Published:** 2023-12-01

**Authors:** Wanglai Li, Huizhang Shen, Zhangxue Huang, Hanzhe Yang

**Affiliations:** Antai College of Economics and Management, Shanghai Jiao Tong University, Shanghai 200030, China; lwl1411@sjtu.edu.cn (W.L.); 2.7182818284@sjtu.edu.cn (Z.H.); dhyana@sjtu.edu.cn (H.Y.)

**Keywords:** rumor, public opinion, nonlinear oscillator model, chaotic behavior

## Abstract

In public opinion triggered by rumors, the authenticity of the information remains uncertain, and the main topic oscillates between diverse opinions. In this paper, a nonlinear oscillator model is proposed to demonstrate the public opinion triggered by rumors. Based on the model and actual data of one case, it is found that a continuous flow of new information about rumors acts as external forces on the system, probably leading to the chaotic behavior of public opinion. Moreover, similar features are observed in three other cases, and the same model is also applicable to these cases. Based on these results, it is shown that our model possesses generality, revealing the evolutionary trends of a certain type of public opinion in real-world scenarios.

## 1. Introduction

Public opinion is the collective opinion on a specific topic or voting intention relevant to society. It reflects the perspectives and attitudes of people on matters that have effects on their lives. With the development of Internet technology, more and more people choose to obtain information and express their opinions through online social media. The channels for spreading public opinion have also gradually shifted from offline to online. However, the wide range and scale of the Internet has led to a variety of online information that is difficult to classify as true and false. This unconfirmed information is called rumor [1].

If rumors are further spread, more netizens will discuss the related topics intensely, and an endless stream of information will emerge. Thus, public opinion is formed, which may evolve into an online group event. In the process of rumor propagation, the truth can be distorted, and social stability can be threatened. Therefore, it is urgent and important to crack down on rumors.

Many studies have been undertaken, which look at rumor propagation and the dynamical behavior of public opinion triggered by rumors on online social networks. Daley [2] analyzed the similarity between epidemics and rumors using mathematical analysis. Then, Daley and Kendal [3] proposed a classical model, the D-K model. In the D-K model, people were categorized into three groups: ignorants, spreaders and stiflers. Until now, epidemic models and their variants have been widely used to simulate the spread of rumors [4]. Chen et al. [5] proposed a novel rumor-spreading model with latent, constant recruitment and varying total population. Soodeh Hosseini et al. [6] studied rumor spreading through a variant of the SEIR epidemic model. Dong et al. [7] constructed an improved XYZ–ISR two-layer model considering time delay to describe the dynamic process of rumor propagation in multiple channels.

In addition, some scholars also used some theories and models in physics to study the phenomenon and nature of rumors, such as the Ising model, complex systems theory, self-organization theory and so on. These were studies of sociophysics. Physicists usually see the big picture and employ approximations that simplify problems while still considering the key processes involved [8]. Methods of physics are enhancing our understanding of society [9]. Li et al. [10] constructed a social network rumor propagation dynamics model and then revealed the rumor transmission rules based on the Ising model. Ma et al. [11] drew the rumor-spreading threshold by means of the generation function and cavity method developed from statistical physics of disordered systems. Pan and Shen [12] found that the diffusion system of a mass incident in Weibo satisfied Power Law, Zipf’s Law, 1/f noise and self-similarity, which meant the system was a self-organization criticality system. Cheng et al. [13] constructed a thermal radiation model of network rumor propagation in the analogy of the thermal radiation process.

Oscillation is a physical phenomenon, which refers to the repetitive variation in time or between some different states. It is also commonly observed in society, such as business cycles in economics [14], population cycle in human societies [15] and others. In public opinion, the presence of diverse perspectives may also lead to a seemingly oscillating pattern, as it appears to shift back and forth between different viewpoints. Some studies of sociophysics introduced oscillator models to study public opinion. Adil Imad Eddine Hosni et al. [16] found that the attraction of an individual to a rumor was similar to an oscillator system when it was displaced from its equilibrium position. Dong et al. [17] proposed a damped oscillator model (DOM), which could predict online public opinion.

To study oscillations in public opinion, the types and features of oscillation need to be further reviewed. Within the field of physics, there are several types of oscillations, including damped oscillations, driven oscillations, and others. Similar patterns of oscillations can be observed in public opinion. If there is no more new information, the public opinion will eventually decay to calmness, akin to the behavior of damped oscillations. For example, on 7 June 2022, an online rumor surfaced claiming that the questions in the Chinese National College Entrance Examination had been leaked. This triggered a heated discussion among netizens and generated public opinion. The next day, the Ministry of Education issued a statement denying the existence of any leaked questions and confirming that the rumor was false. Subsequently, there was no new information on this topic, and the public opinion gradually decayed to calmness.

However, the continuous releasing of new information may lead to continuous oscillations of public opinion, akin to the behavior of driven oscillations. Some oscillations may lead to the occurrence of complex behaviors, especially in public opinion triggered by rumor.

In this paper, a nonlinear oscillator model is proposed to demonstrate the public opinion triggered by rumors. This model can effectively reflect the situations where the authenticity of the information remains uncertain, and the main topic oscillates between diverse opinions. Based on the model, chaotic behavior in rumor propagation is identified.

The remaining structure of this paper is organized as follows. In Section 2, we first introduce the driven damped pendulum model in physics. In Section 3, a comparison between rumor propagation and the swing of driven damped pendulum is conducted, and a nonlinear oscillator model for public opinion is proposed. In Section 4, a real case is analyzed to determine values of each parameter in the model, so the model can reflect the evolution of this case. In Section 5, chaotic behavior and chaos control for this model are explored. In Section 6, based on the findings above, three current cases are analyzed, which confirm the generality of this study. In Section 7, the entire research process is summarized, and an outlook on future research directions is presented.

## 2. Driven Damped Pendulum Model

During the process of rumor propagation, both resistance to their spread and a continuous flow of new information being released are present. Therefore, certain similarities exist between the rumor propagation and the swinging of a driven damped pendulum.

**Figure 1:**
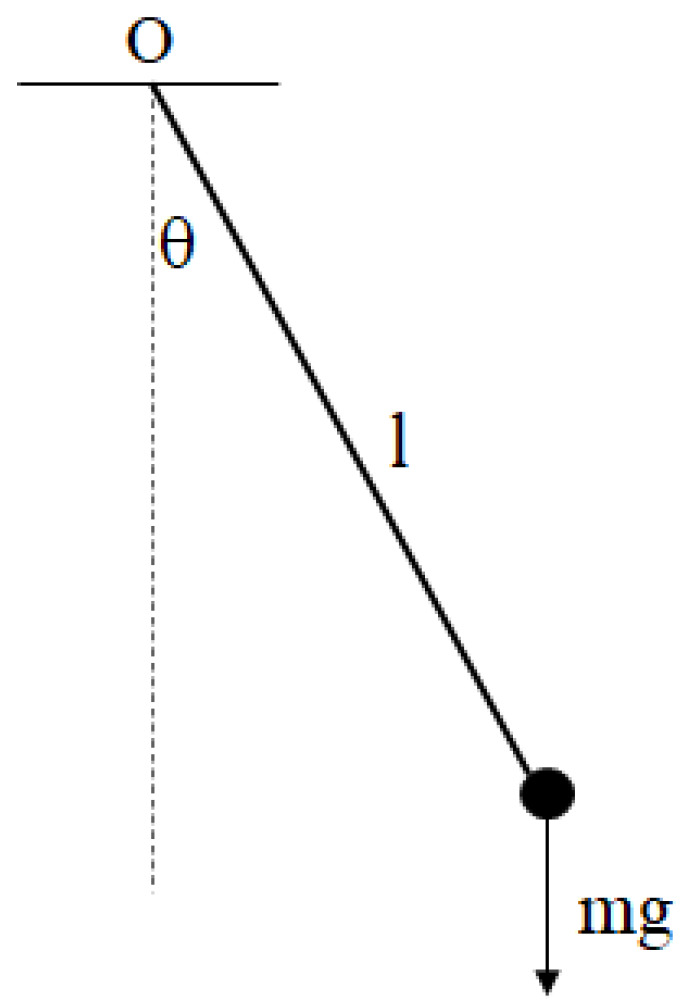
A driven damped pendulum.

The driven damped pendulum is one of the most classic models in physics, which includes a rod and a bob attached to the end of the rod, as shown in Figure 1. The length of the rod is l, and the mass of the bob is m. The angle that the pendulum swings away from vertical is θ, which is also called angle of the pendulum. This system seems to be simple but, in fact, it is a complex nonlinear system, which can produce a variety of complex behaviors according to existing studies [18,19].

The model satisfies the following conditions:

(1) The rod is massless, and the mass of the whole system is concentrated on the bob.
(2) The rod is rigid, so the angle θ can exceed 90°, approach or reach 180° or even exceed 180°.
(3) The bob is subject to tangential resistance when swinging, and the magnitude of the resistance is $\gamma l\frac{d\theta }{dt}$
(4) The bob is subjected to periodic external forces in the direction of oscillation, which is always perpendicular to the pendulum. Some books and papers set up external forces to satisfy the cosine form [20], which is $F\times \cos\left(\omega_{D}t\right)$.

Therefore, the dynamical equation can be written as:

(1) $$ml\frac{d^{2}\theta }{dt^{2}}+\gamma l\frac{d\theta }{dt}+mg\sin\theta =F\times \cos\left(\omega_{D}t\right)$$

When θ is relatively small, i.e., sin⁡θ≈θ, and F=0, the motion of the pendulum can be reduced to simple harmonic oscillation. But when θ is larger, the nonlinear part of the equation cannot be ignored, and a variety of complex dynamical behaviors may evolve.

In the study of its properties, it is common to use a dimensionless treatment for the above equation. This treatment simplifies the dynamical equation, reduces the number of parameters involved, and implicitly incorporates other influencing factors.

(2) $$\frac{d^{2}\theta }{d{\left(\tau \right)}^{2}}+2\beta \frac{d\theta }{d\tau }+\sin\theta =f\times \cos\left(\Omega \tau \right)$$

where $\omega_{0}=\sqrt{g/l},\beta =\gamma /\left(2m\omega_{0}\right),f=F/\left(mg\right),\Omega =\omega_{D}/\omega_{0},\tau =\omega_{0}t$.

## 3. The Nonlinear Oscillator Model for Public Opinion Triggered by Rumors

Through analogical analysis, each feature in rumor propagation can be demonstrated by the pendulum model. For example, the direction of public opinion can be analogous to the angle of the pendulum, the heat of public opinion can be analogous to the energy of the pendulum system, and so on. However, there are also some differences between them. For example, the common functional form of periodic external forces in the pendulum model is the cosine force, while in the real world, it is difficult to find the cosine form of external forces.

Therefore, the dynamical equation of the driven damped pendulum model is adjusted as follows:

(3) $$\frac{d^{2}\theta }{dt^{2}}+2\beta \frac{d\theta }{dt}+\sin\theta =f\times h\left(t\right)$$

where $h\left(t\right)$ is a periodic function of the external forces. Periodic external forces play an important role in the evolution of the nonlinear system. The function form of $h\left(t\right)$ will be illustrated in Section 3.5.

This dynamical equation is the nonlinear oscillator model for public opinion triggered by rumors. The state variables, parameters and functions in Equation (3) are given a sociological interpretation as follows.

### 3.1. Direction of Public Opinion—Angle of the Pendulum

In public opinion triggered by rumors, there are typically two types of opinions in general, which can also be referred to two directions of public opinion. One represents the opinions that support or believe the rumors, while the other represents the opinions that oppose the former one and aim to crack down on rumors. This categorization for opinions is justified in real-world situations and finds extensive usage in various academic studies. In Equation (3), it can be illustrated by the angle of the pendulum θ. The positive and negative signs in θ correspond to the type of opinions. And $\frac{d\theta }{dt}$ corresponds to the speed of the oscillations in public opinion, essentially reflecting the intensity of the argument between different opinions.

### 3.2. Heat of Public Opinion—Energy

The heat of public opinion describes the level and impact of public opinion. The more netizens participate in the discussion, and the wider the spread, the higher the heat of public opinion becomes. Through analogical analysis, the heat of public opinion is akin to the energy of the pendulum system. Therefore, the energy formula of the pendulum system can be used to measure the heat of public opinion, which is as follows, combining gravitational potential energy and kinetic energy.

(4) $$E\left(t\right)=1-\cos\theta +0.5\omega^{2}$$

where $\omega =\frac{d\theta }{dt}$.

Since the dynamical equation is dimensionless, the energy Formula (4) is dimensionless, too.

### 3.3. Interaction between Different Opinions—Gravity

When there are no external forces or damping acting on it, public opinion still oscillates spontaneously. This is because public opinion is shaped by diverse individuals who hold different opinions and seek to persuade or refute others. These interactions between different opinions behave as an internal force and make public opinion fluid and dynamic, resulting in a constantly evolving direction of public opinion that can change over time.

As the pendulum swings back and forth due to gravity, the internal force that drives this evolution of public opinion is akin to gravity. In Equation (3), the corresponding part is sin⁡θ.

### 3.4. Resistance to Spread—Damping

In addition to the above internal force, during the evolution of public opinion triggered by rumors, there exists resistance to control the spread of relevant information. For example, some management departments may take measures such as deleting posts, and concerned organizations may engage in fact-checking. If there is no more new information, the public opinion will eventually decay to calmness under the influence of resistance. 

In the pendulum system, the energy is dissipated from the system as a result of damping. If no external force is applied, the pendulum eventually tends to return to the equilibrium position and stop. Therefore, resistance to spread in public opinion corresponds to the damping in the pendulum. The value of β indicates the degree of resistance.

### 3.5. New Information or Energy—External Force

During the evolution of public opinion, some new information can be released at some moments, including but not limited to the exaggeration of rumors, disinformation, news reports, responses to the rumor and more. The information serves as an energy input to public opinion, which results in continuous oscillations of public opinion.

In the pendulum system, the input of energy is achieved through the work carried out by the external force. External force is a crucial factor in triggering the complex behavior of the pendulum system [21]. With the presence of continuous and periodic external forces, the system may evolve into more complex behaviors over time.

Therefore, continuous new information or energy in public opinion is expressed as periodic external forces in the model. Two directions of external forces are related to two directions of public opinion. One is to deepen the degree of believing and spreading rumors, while the opposite is to dispel rumors. The external forces alternate in two directions and follow a periodic pattern.

External forces may have either positive or negative feedback effects on the system’s energy. For example, the spread of rumors will amplify the impact on public opinion, which is a positive feedback effect; but, if the information is excessively exaggerated, netizens may become skeptical and choose not to believe it, resulting in a negative feedback effect. Prompt response to rumors may calm down public opinion, which is a negative feedback effect; but, an inappropriate response will fuel public opinion and produce a positive feedback effect. As a result, the heat of public opinion in our model also shows the pattern of continuous oscillations, which is basically consistent with reality.

In order to determine the functional form of periodic external forces, $h\left(t\right)$ in the nonlinear oscillator model needs to be explored. As mentioned before, the cosine function is unrealistic and not the right choice. Here, we would like to introduce the concept of “information obsolescence”. Information obsolescence refers to the phenomenon where the value of information decays over time, regardless of its form, such as a document, web page or other format. It means that information may become valueless or outdated due to changing circumstances. Recent studies suggest that information obsolescence is also observed on social media platforms, where the value of information decays as it spreads [22].

The functional form of $h\left(t\right)$ should include information obsolescence, and the process of information obsolescence is mathematically expressed as a negative exponential function of time.

(5) $$h\left(t\right)=\left\{\begin{array}{c} e^{-\alpha \left(t-nT\right)}\;\;\;\;\;\;nT\leq t<nT+0.5T\\ -e^{-\alpha \left(t-nT-0.5T\right)}\;\;nT+0.5T\leq t<\left(n+1\right)T \end{array}\right.$$

where α denotes the decay rate of information, T denotes the period of external force and the minimum interval between positive and negative external forces is T/2. 

## 4. Numerical Experiment Based on One Case

The change of parameters in the dynamical equation will cause a great change in the state of the system corresponding to the dynamical equation, so the study of the parameters in the nonlinear dynamical equation is very important to understand and grasp the propagation and evolution of rumors.

In this section, the “Death of Wang Fengya” case that occurred in 2018 is used for the numerical experiment. The values of each parameter are determined based on the evolution of public opinion. This lays the foundation for the next section in studying the long-term evolutionary trends of the system.

### 4.1. Case Brief

Wang Fengya, a young girl born in 2015 in Henan, was diagnosed with retinoblastoma in 2017 and died on 4 May 2018. On 24 May 2018, an article titled “Death of Wang Fengya” was published, detailing how Wang Fengya’s family had raised CNY 150,000 via a social fundraising platform called shuidichou. Shockingly, the article revealed that the funds were not used for Wang Fengya’s tumor treatment but for her brother’s harelip treatment instead. The information rapidly gained attention from netizens, who expressed suspicions about the family’s motives and raised questions about the legitimacy of this fundraising campaign. Public opinion was began to evolve. However, Shuidichou confirmed that the actual amount raised was only CNY 35,000, significantly less than the CNY 150,000 rumored on the internet. Furthermore, after an investigation, the local police stated that the majority of the funds raised were indeed used for Wang Fengya’s treatment. Another foundation also clarified that they had covered the cost of Wang Fengya’s brother’s harelip treatment, and there was no misuse of the donations by the family. Wang Fengya’s grandfather disclosed the donation details and donated the remaining CNY 1301 to the local charity association. Nevertheless, there were still many netizens discussing this case. The oscillations of public opinion persisted for a long period of time.

### 4.2. Model Verification

“Zhiweidata” is a famous comprehensive data analysis platform of online events in China and was widely used in research [23]. “Zhiweidata” can offer data of spread trends for each case. These data demonstrate the quantity of relevant messages posted on social media platforms during a period of time, such as Weibo, WeChat and other online media. Therefore, the “spread trend” data from the “Zhiweidata” platform are used to measure the heat of public opinion.

**Figure 2:**
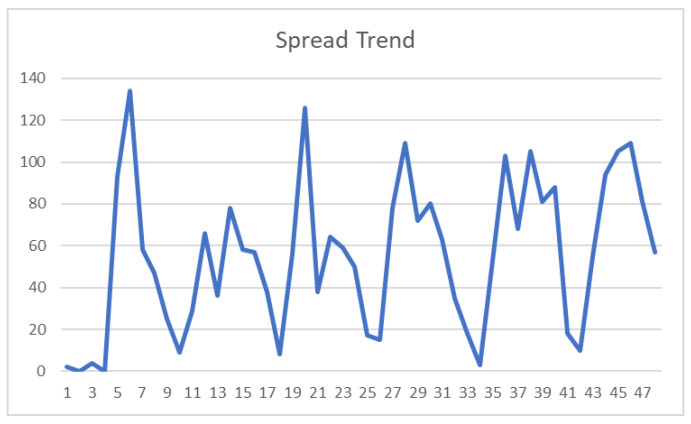
Real spread trend of the “Death of Wang Fengya” case.

Hourly data for “Death of Wang Fengya” were collected from 24 May 2018 to 29 May 2018. In order to make the spread trend curve smoother, the data were aggregated by calculating the sum of the propagation data every 3 h, resulting in a total of 48 data points. To improve clarity in presentation, the following figure displays the propagation trend with an index ranging from 1 to 48 instead of using specific time intervals, as shown in Figure 2.

**Figure 3:**
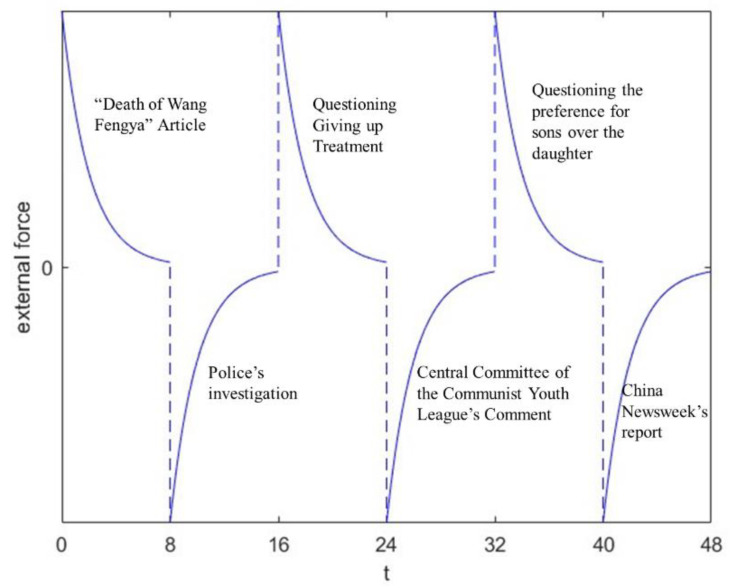
Correspondence between external force trends and new information in the case of Wang Fengya’s death.

As can be seen from Figure 2, there were lots of discussions about the “Death of Wang Fengya” and the heat of public opinion underwent continuous oscillations, resulting in the formation of daily energy peaks. This is closely related to the action of new information every day. Hence, the curve representing the external forces in the simulation should assume the shape depicted in Figure 3. On each day, it is also labeled with new information in reality.

On 24 May, an article titled “Death of Wang Fengya” was spread on social media. The article revealed that the funds were not used for Wang Fengya’s tumor treatment but for her brother’s harelip treatment instead. The information raised doubts about the motives of the family and the legitimacy of this fundraising event. This was an external force in the positive direction.

However, on 25 May, after an investigation, the police confirmed that there was no misappropriation of the donation. Wang Fengya’s grandfather also disclosed the details of the donation and announced that the remaining funds would be donated to local charities. This was an external force in the negative direction.

On 26 May, an article was posted that Wang Fengya’s mother had given up treatment several times. Additionally, the article pointed out that some of donations were used for purchasing toys and milk powder, which led netizens to question whether these things were for Wang Fengya. This was an external force in the positive direction.

On 27 May, the Central Committee of the Communist Youth League commented on this case and called for rational thinking based on facts. This was an external force in the negative direction.

On 28 May, some pointed out that the preference for sons in the family was the cause of Wang Fengya’s tragedy, as both her grandfather and parents were more eager to have a boy. This was an external force in the positive direction.

Finally, on 29 May, China Newsweek published an article to summarize the incident, clarify many rumors and expose the behavior of those who made false claims on social media. This was an external force in the negative direction.

In order to reflect the reality mentioned above, as for the dynamics model, the parameters shown in Table 1 are taken.

**Figure 4:**
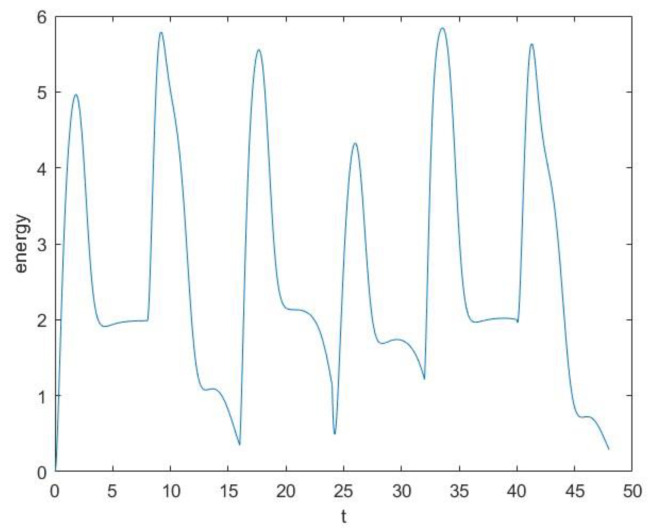
Simulation spread trend of the model.

Under the value of parameters, the Runge–Kutta method is utilized to solve the model, and the simulation data are illustrated in Figure 4. The horizontal axis represents time, while the vertical axis represents the energy of the dynamical system, corresponding to the heat of public opinion.

The actual data and simulation data do not have the same magnitudes and intervals, and the scale of public opinion is not the focus of our research. Its emphasis should lie on studying the patterns of public opinion oscillation rather than comparing their absolute values. So, only the relative evolutionary trends of the two are compared.

As shown in Figure 2 and Figure 4, the actual spread trend and the simulation spread trend are basically similar from a macroscopic perspective. Then, dynamic time warping (DTW) is used to measure their similarity. In time-series analysis, DTW is an algorithm for measuring similarity between two temporal sequences [24]. Because our focus is solely on relative evolutionary trends, both the actual data and simulation data need to be normalized by min–max normalization before computation. As a result, the values are scaled and confined within the range of $\left[0,\;1\right]$. The DTW analysis yields a final result of 0.1130, indicating there is a difference of 0.1130 between the reality and the model for each data point on average. Therefore, the numerical result indicates that the model can reflect reality.

## 5. Analysis of Long-Term Evolutionary Trend

### 5.1. Chaos

Chaos is an important concept in systems science. Chaotic behavior can be observed in various dynamical systems, such as fluid dynamics, weather patterns, and climate. If a dynamical system is classified as chaotic, it must be sensitive to initial conditions. This means that even small changes in the initial conditions can lead to significant differences in the system’s evolution.

From the above analysis, the parameters of the model are determined, and the validity of the model is verified. Then, for the dynamical system corresponding to the model, its dynamical behavior is explored by phase portrait and largest Lyapunov exponent.

(1) Phase Portrait

**Figure 5:**
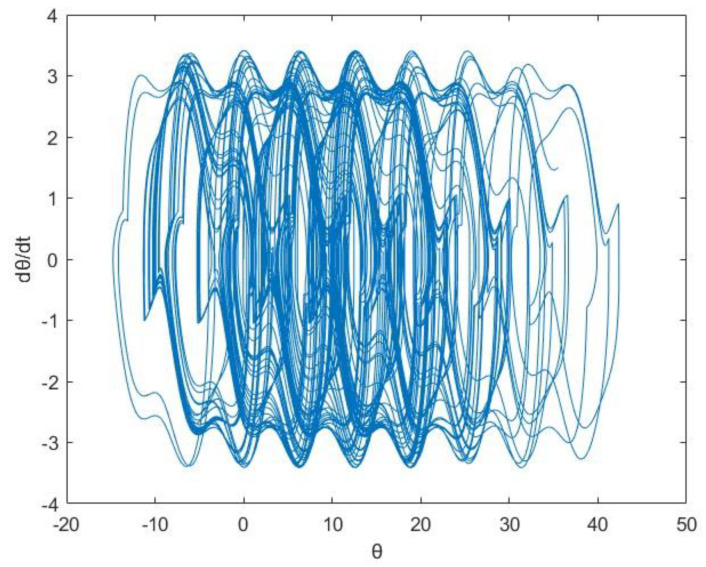
Phase portrait of $\left(\theta ,\frac{d\theta }{dt}\right)$.

In mathematics, a phase portrait is a geometric representation of the trajectories of a dynamical system in the phase plane. Phase portraits are an invaluable tool in studying dynamical systems. In the following, we utilize the phase portrait depicted in Figure 5 to depict the orbit of $\left(\theta ,\frac{d\theta }{dt}\right)$ and describe the nonlinear oscillator motion in the given case.

(2) Largest Lyapunov Exponent

Being sensitive to initial conditions is one of the characteristics of a system in a chaotic state. The Lyapunov exponent of a dynamical system is a quantity that characterizes the rate of separation of infinitesimally close trajectories. A positive largest Lyapunov exponent (LLE) is usually taken as an indication that a system is chaotic. Therefore, it is sufficient to calculate its largest Lyapunov exponent $\lambda_{1}$ in this paper.

There are lots of algorithms to calculate LLE. In this paper, G. Benettin’s algorithm [25] is used. Almost all methods for the computation of Lyapunov Exponents are more or less based on G. Benettin’s research [26]. First, track the evolution of two close points on the attractor after a short period of time, and calculate the LLE value approximately for this stage using the duration and the amplitude of disturbance. Then, renormalize the points and repeat the described procedure. After a certain number of repetitions, we find the final LLE as the arithmetic mean of the values obtained at each stage [27].

The LLE is as follows:

$$\lambda_{1}\approx 0.0908>0$$

According to the phase portrait and the largest Lyapunov exponent, the public opinion of this case can generate chaotic behavior.

### 5.2. Chaos Control

Chaos control is to render amotion more stable and predictable instead of chaotic. A feedback control method utilizing negative feedback is proposed, and the associated dynamical equation is presented below.

(6) $$\frac{d^{2}\theta }{dt^{2}}+2\beta \frac{d\theta }{dt}+\sin\theta =f\times h\left(t\right)-k\frac{d\theta }{dt}{\left(E\left(t\right)\right)}^{2}$$

where $E\left(t\right)$ denotes the energy of the system, and *k* denotes the intensity of control measures. In this paper, k=0.5.

The control method corresponds to actions taken by the organization to manage rumors, which are as follows:

(1) Firstly, the oscillating direction and heat of public opinion need to be accurately monitored;
(2) Actions to control the rumor are customized based on the oscillating direction and heat of public opinion. For example, the direction to guide public opinion should be opposite to the oscillating direction of public opinion so that negative feedback can be established; The higher the heat of public opinion, the greater the force of guidance.

**Figure 6:**
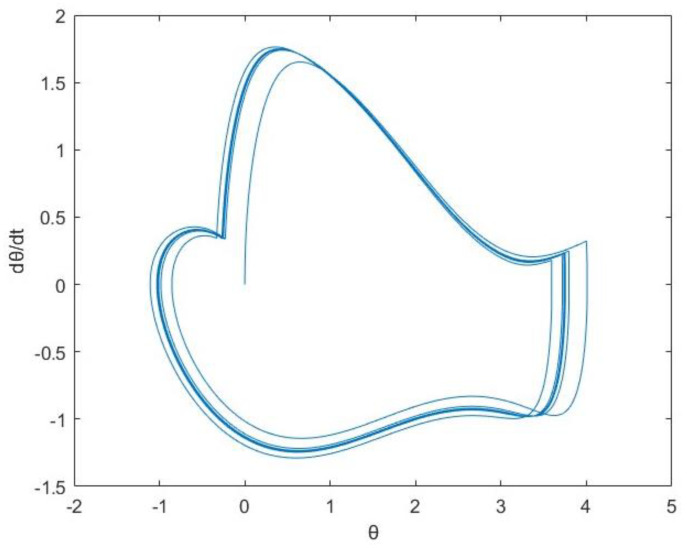
Phase portrait under chaos control.

Using phase portrait and largest Lyapunov exponent, a test is carried out to determine whether the nonlinear system can exit the chaotic state following the implementation of chaos control. The phase portrait is shown in Figure 6.

The LLE is as follows:

$$\lambda_{1}\approx -0.0522<0$$

According to the phase portrait and the largest Lyapunov exponent, through chaos control, the nonlinear system is successfully made to get rid of the chaotic state by controlling the impact of external forces on the system.

### 5.3. Summary

Based on the above results, in the “Death of Wang Fengya” case, the continuous flow of new information acted as external forces, leading to the chaotic behavior of public opinion. Consequently, this led to complex oscillations in public opinion and a confusing blend of accurate and inaccurate information. By controlling the impact of new information, it would be possible to prevent the occurrence of chaotic states.

## 6. Generality of the Model

The chaotic behavior of “Death of Wang Fengya” case is found through the nonlinear oscillator model. It is necessary to explore whether the nonlinear oscillator model can reflect the situations of other cases. Therefore, in this section, the generality of the model will be confirmed.

Firstly, three cases are selected and briefly described. The details of each case are shown as follows.

(1) Multiple violations exposed against Hengshui Taocheng Middle School

This case originated from an allegation against Hengshui Taocheng Middle School, China. Some netizens disclosed various violations, including the school’s violations in organizing exams, the employment of abusive practices such as corporal punishment and other things. In addition, there was an allegation that a teacher at the school molested students. On 16 February 2022, the relevant topics swiftly spread on social media, and lots of netizens voiced strong criticism against the school. On 17 February, the local education bureau declared its ongoing investigation into the allegations. During the evolution of public opinion, a large amount of unverified information emerged online, making it difficult to identify its authenticity. Up to 21 February, local police stated that the teacher had not exhibited any indecent behavior. However, on 22 February, an official investigation report showed that some of the issues complained about truly happened, and the school did not pass its annual inspection in 2022.

(2) Forestry owners begging for water in a kneeling position

In late March 2023, a video went viral on social media, depicting a man kneeling on the sand and pleading for water to plant trees after his water source was destroyed by a nearby mine. A significant number of netizens expressed their support and sympathy for the man, Mr. Sun, lauding him as a desertification control hero. After that, the two sides did not reach an agreement on the water supply during the dispute. Meanwhile, many condemned the mine and the local government. However, as public opinion evolved, some netizens discovered that the trees planted by Mr. Sun were not typical anti-desertification vegetation but rather cash crops. This revelation led to questioning and criticism of Mr. Sun’s actions.

(3) Strange object in the college canteen

This case originated from a video claiming to have found a strange object resembling a rat’s head in the canteen food of a college in Jiangxi, China. However, on 3 June 2023, the concerned college stated that the “strange object” was a duck neck. This statement from the school ignited further discussion. Some netizens believed it was a duck neck, while others thought the response was unconvincing, ridiculing it as “calling a mouse a duck”. The situation escalated with endless online information, resulting in chaos. It was challenging to differentiate between what was true and what was a rumor. On 10 June, a joint investigation by several departments, including the Education Department of Jiangxi Province, was conducted. After a few days, it was confirmed that the “strange object” was indeed made from the head of a rodent resembling a mouse.

There was a continuous flow of new information in each of these three cases, and the conflicting information drove the intense oscillation of public opinion, indicating that they exhibited similar features to the “Death of Wang Fengya” case. As a result, the parameters used for these cases are the same as those listed in Table 1.

Then, the similarities between the actual data of these cases and the simulation data of the model are measured. In the same way as the preceding steps in Section 4, the actual data are also obtained from the “Zhiweidata” platform, and both the actual data and simulation data need to be normalized by min–max normalization before computation. The final results of the DTW analysis are presented in Table 2.

These values suggest that the model can reflect the reality of these cases. In these cases, the new information acted as external forces to make the system descend into a state of chaos. That is, the focus of online public opinion is constantly changing, making it difficult to predict the main topics in the next stage; mixed and unverified online information hinders authenticity. Based on these results, it is shown that our model possesses generality and describes the evolutionary trends of a certain type of public opinion in real-world scenarios.

## 7. Conclusions

In this paper, a nonlinear oscillator model is proposed to study the dynamical behavior of public opinion triggered by rumor. It is found that chaotic behavior may result from a continuous flow of new information. However, there are also limitations to the model. The model is constructed from a macroscopic perspective, and individual-level details are simplified. Consequently, the model’s capacity is restricted to capturing macro-level trends while lacking precision in describing specific details. In the future, there is potential to enhance this research from a microscopic perspective. This can be achieved by employing a multi-agent modeling method to explore how information affects each individual and how interactions between individuals within crowds influence the evolution of public opinion.

## Figures and Tables

**Table 1:** Parameters of the model.

Parameters	Value
T	16
β	0.45
f	5.5
α	0.5

**Table 2:** Similarities between each case and the model.

Case	Time Period	Average Difference
Multiple violations exposed against Hengshui Taocheng Middle School	16 February 2022–22 February 2022	0.1383
Forestry owners begging for water in a kneeling position	28 March 2023–2 April 2023	0.1030
Strange object in the college canteen	3 June 2023–10 June 2023	0.1201

## Data Availability

The data and code presented in this study are available on request from the corresponding author.
